# Hypoxia-sensitive miRNA regulation *via* CRISPR/dCas9 loaded in hybrid exosomes: A novel strategy to improve embryo implantation and prevent placental insufficiency during pregnancy

**DOI:** 10.3389/fcell.2022.1082657

**Published:** 2023-01-10

**Authors:** Alireza Yaghoobi, Yasaman Nazerian, Arman Zeinaddini Meymand, Ali Ansari, Amirhossein Nazerian, Hassan Niknejad

**Affiliations:** ^1^ Department of Pharmacology, School of Medicine, Shahid Beheshti University of Medical Sciences, Tehran, Iran; ^2^ School of Medicine, Iran University of Medical Sciences, Tehran, Iran

**Keywords:** infertility, recurrent implantation failure, endometrial receptivity, mir-30d, HIF-1α, liposomeexosome hybrid, CRISPR/dCas9, regenerative medicine

## Abstract

Assisted reproductive techniques as a new regenerative medicine approach have significantly contributed to solving infertility problems that affect approximately 15% of couples worldwide. However, the success rate of an *in vitro* fertilization (IVF) cycle remains only about 20%–30%, and 75% of these losses are due to implantation failure (the crucial rate-limiting step of gestation). Implantation failure and abnormal placenta formation are mainly caused by defective adhesion, invasion, and angiogenesis. Placental insufficiency endangers both the mother’s and the fetus’s health. Therefore, we suggested a novel treatment strategy to improve endometrial receptivity and implantation success rate. In this strategy, regulating mir-30d expression as an upstream transcriptomic modifier of the embryo implantation results in modified expression of the involved genes in embryonic adhesion, invasion, and angiogenesis and consequently impedes implantation failure. For this purpose, “scaffold/matrix attachment regions (S/MARs)” are employed as non-viral episomal vectors, transfecting into trophoblasts by exosome-liposome hybrid carriers. These vectors comprise CRISPR/dCas9 with a guide RNA to exclusively induce miR-30d gene expression in hypoxic stress conditions. In order to avoid concerns about the fetus’s genetic manipulation, our vector would be transfected specifically into the trophoblast layer of the blastocyst *via* binding to trophoblast Erb-B4 receptors without entering the inner cell mass. Additionally, S/MAR episomal vectors do not integrate with the original cell DNA. As an on/off regulatory switch, a hypoxia-sensitive promoter (HRE) is localized upstream of dCas9. The miR-30d expression increases before and during the implantation and placental insufficiency conditions and is extinguished after hypoxia elimination. This hypothesis emphasizes that improving the adhesion, invasion, and angiogenesis in the uterine microenvironment during pregnancy will result in increased implantation success and reduced placental insufficiency, as a new insight in translational medicine.

## 1 Introduction

Infertility has consistently emerged as a worldwide couple’s problem. Today, up to 15% (nearly 50 million) of all reproductive-aged couples face infertility (Obstet. Gynecol, 2020; Zhang et al., 2022), and this number seems to be rising because of postponing the pregnancy (Bouzaglou et al., 2020; Glick et al., 2021). As a result, for the past four decades, 40 million patients have sought infertility treatments known as assisted reproductive technology (ART), and 7 million neonates have been born *via in vitro* fertilization (IVF) (Shi et al., 2017; Kuroda et al., 2018). However, despite the rapid developments in IVF techniques, the possibility of a successful gestation remains 20%–30% in each IVF cycle. Seventy-five percent of these losses are due to implantation failure as the crucial rate-limiting step of gestation (Niederberger et al., 2018). Meanwhile, some women undergo repeated IVF cycles without establishing a clinical pregnancy, a condition diagnosed as recurrent implantation failure (RIF) (Coughlan et al., 2014; Makrigiannakis et al., 2021). Regardless of the underlying disease, impairments in each phase, including adhesion, invasion, and angiogenesis, could lead to implantation failure (Coughlan et al., 2014; Moreno-Moya et al., 2014).

Adhesion, invasion, and angiogenesis take place in the context of bidirectional embryo-maternal communication. This communication occurs within the complex molecular signaling pathways, consisting of adhesion molecules, cytokines, chemokines, and growth factors which are derivatives of either the embryo or the endometrium (Vilella et al., 2015; Balaguer et al., 2019; Fukui et al., 2019). These coordinated series of actions eventually form an appropriate uterine microenvironment, recognized as receptive endometrium (Thouas et al., 2015; Salamonsen et al., 2016). Endometrial receptivity is mainly regulated by hormonal responses and mutual molecular communication between the embryo and the endometrium, whereas it was previously assumed that the hormonal reactions are solely responsible for preparing the receptive endometrium (Hart et al., 2022).

After appreciating the critical role of molecular communication in endometrial receptivity, extracellular vesicles (EVs) were investigated as novel intercellular communication tools. EVs can transfer different macromolecules such as nucleic acids (DNA, mRNAs, and microRNAs), lipids, and proteins between cells (Governini et al., 2021). In between, microRNAs are considered one of the key regulators of gene expression during implantation (Andronico et al., 2019; Hart et al., 2022). MicroRNAs (miRNAs) are 18–25 non-coding nucleotide chains that regulate post-transcriptional gene modifications and participate in various intercellular interactions as well as embryo-maternal cross-talk during implantation (Galliano and Pellicer, 2014; Liang et al., 2017). miRNAs are mostly incorporated within the exosomes and are assumed to be crucial bioactive molecules for blastocyst implantation and embryo-maternal communications during embryo development (Bridi et al., 2020).

Amongst miRNAs, the miR-30d role has been investigated in human’s uterus biopsy (Liang et al., 2017; Bridi et al., 2020). miR-30d is an upstream signaling molecule, and its upregulation results in the increased expression of endometrial estrogen and progesterone receptors, LIF in uterus and trophoblast, integrating and adhesive molecules including integrin alpha-7 (ITGA7), integrin beta-3 (ITGB3), cadherin-5 (CDH5) as well as COX2 enzyme. Eventually, the upregulation of these signaling and structural molecules enhances endometrial receptivity and ameliorates blastocyst adhesion, invasion, and angiogenesis (Vilella et al., 2015; Liang et al., 2017; Balaguer et al., 2019; Timofeeva et al., 2019). In 2019, (Balaguer et al., 2019) used mice models to evaluate the impact of miR-30d deficiency on pregnancy. They demonstrated that either maternal or embryonic miR-30d insufficiency leads to diminished implantation, placentation, and fetal growth (Balaguer et al., 2019).

The potential role of microRNA-30d in RIF patients has been discussed in previous studies. RIF patients have lower miR-30d expression levels, increased SOCS1 expression levels, lower endometrial and blood levels of LIF, and downregulated JAK-STAT3 pathway (Altmäe et al., 2013; Arias-Sosa et al., 2018). miR-30d can inhibit SOCS1, which consequently increases LIF levels and activates the JAK-STAT3 pathway. So, miR-30d seems to improve endometrial receptivity as one of the major causes of implantation failure (Altmäe et al., 2013; Lin et al., 2017).

To overcome ART challenges, we proposed a novel strategy to upregulate miR-30d expression as an upstream signaling molecule to increase the probability of a successful gestation by improving blastocyst adhesion, invasion, and angiogenesis as well as keeping the forthcoming fetus alive during pregnancy. In brief, the proposed strategy includes designing an episomal vector based on a scaffold/matrix attachment region (S/MAR) that mainly consists of CRISPR/dCas9 with a guide RNA, hypoxia sensitive promoter (HIF-1 sensitive), and doxycycline sensitive on/off switch. The vector will be transferred to the blastocyst trophoblast specifically by engineered exosomes to avoid concerns about the fetus’s genetic manipulation. Then, the blastocyst is transferred to the uterus *via* IVF.

## 2 Supporting evidences for strategy

### 2.1 Endometrial receptivity and implantation

Implantation is considered a bidirectional interaction between the embryo and the endometrial surface, including adhesion, invasion, and angiogenesis. This complex process occurs over approximately a 3–5-day interval during receptivity of the endometrium. This phase is essential for attaching the trophectoderm layer and subsequent invading and vascularization of the embryo. The limited duration of uterine receptivity for implantation during the mid-luteal phase is defined as the “window of implantation” (WOI), with a specific gene expression profile appropriate for embryo attachment and accommodation. Failure of implantation is the leading cause of pregnancy loss in assisted reproductive technology (ART), and it mainly stems from either impaired endometrial receptivity or low-quality embryos. These two are the pivotal causes of unsuccessful implantation, miscarriage, and RIF in people who undergo IVF (Simon and Laufer, 2012; Lessey and Young, 2019; Enciso et al., 2021). Maternal causes of the RIF include anatomical abnormalities, thrombophilia, infection, genetic alteration, immunological factors, and endometrial receptivity (Simon and Laufer, 2012; Bashiri et al., 2018; Makrigiannakis et al., 2021). Studies have demonstrated that RIF patients suffer from relatively lower endometrial receptivity at the time of embryo transfer (Ruiz-Alonso et al., 2013).

A broad spectrum of etiologies are involved in RIF, but the exact mechanisms are not properly understood (Simon and Laufer, 2012; Bashiri et al., 2018). So, looking for further molecular mechanisms that participate in implantation failure is crucial. Meanwhile, microRNAs (miRNAs) are potential modulators of the involved signaling pathways in embryo implantation (Galliano and Pellicer, 2014; Zhou et al., 2020).

### 2.2 miRNAs and pregnancy

miRNAs are 18–25 non-coding nucleotide chains that act as post-transcriptional gene modifiers. miRNAs are substantially involved in modulating normal cells’ development, including cellular differentiation, proliferation, apoptosis, embryo early development, embryo-endometrial communication, endometrium receptivity, implantation, decidualization, and placenta formation (Galliano and Pellicer, 2014; Kolanska et al., 2021). Studies have shown that, in the bidirectional communication between the embryo and the endometrium, both secrete particular microRNAs, which can be taken up by the other party and influence the implantation process (Liang et al., 2017). These secreted miRNAs are rather stable and accessible in embryo culture and uterine fluid, making them potential non-invasive biomarkers to confirm the embryo quality.

Several studies demonstrated that impaired miRNA expression contributes to RIF. Both overexpressed and under-expressed endometrial miRNA patterns have been reported in the mid-secretory phase of RIF patients compared to fertile women (Revel et al., 2011). The expression of endometrial microRNAs is different in impaired endometrium during WOI. Previous studies have revealed unique irregular expression of 105 miRNAs in individuals with RIF (Shi et al., 2017). There was also a positive correlation between upregulation of these miRNAs and successful implantation 24 h after frozen embryo transfer. Downregulation of miR-198, miR-522, and miR-891a has been shown in implantation failure (Parks et al., 2014). Kuokkanen et al., (2010) have shown higher expression levels of 12 miRNAs, including miR-30d, during the mid-secretory phase of the menstrual cycle (Kuokkanen et al., 2010). Additionally, endometrial and serum expression of miR-203, miR-31, miR-30b, and miR-30d were notably unregulated during the implantation period (Kresowik et al., 2014). Other studies have also shown that microRNA polymorphisms significantly differ between individuals with RIF and fertile controls (Lee et al., 2019). So, miRNAs can be used as a regulator of the implantation process.

### 2.3 miR-30d role in embryo implantation

**FIGURE 1:**
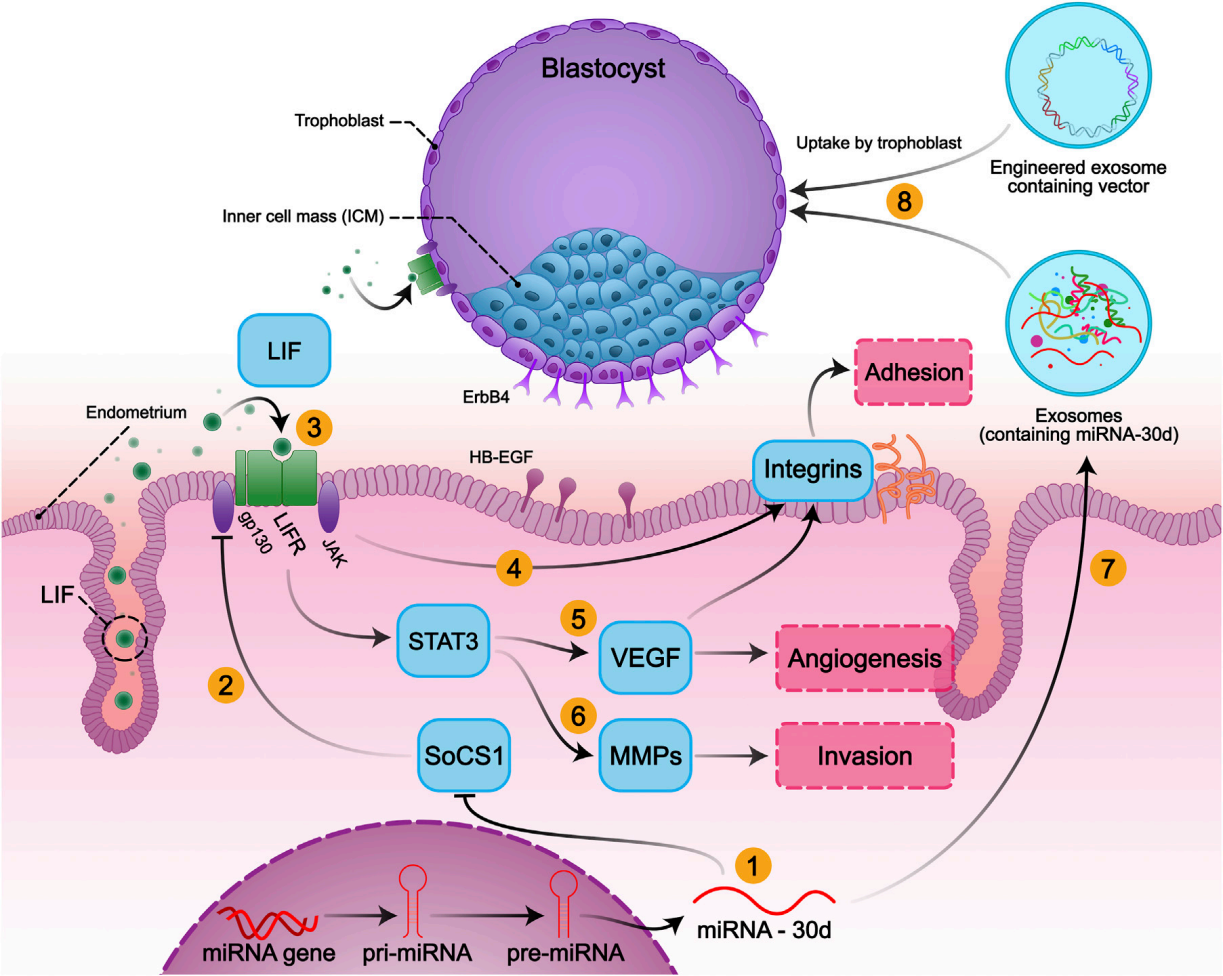
Embryo-Uterine molecular signaling in implantation and the role of miR-30d in embryo implantation: The implantation process involves three main steps: adhesion, invasion, and angiogenesis, which contribute to successful implantation. Expression of mir-30d as a modifier of the embryo implantation modifies its transcriptome, resulting in overexpression of genes involved in embryonic adhesion, invasion, angiogenesis, and endometrial receptivity. (1) MiR-30d promotes the activation of the LIF-induced STAT3 pathway, possibly *via* downregulating SOCS1 in the embryo and endometrium. (2) SOCS1 inhibits the LIF/JAK/STAT3 signaling by inhibiting the activation of JAK. (3) LIF activates gp130/STAT3 signals *via* binding to LIFR and gp130 heterodimer both in endometrium and embryo. Activation of STAT3 (4) induces adhesion by increasing the expression of integrin alpha-7, integrin beta-3, and also (5) promotes angiogenesis by inducing VEGF expression and (6) invasiveness of trophoblastic cells by activating the transcription of MMPs. (7) Human endometrium secretes miR-30d, (8) then exosomal has-miR-30d is taken up by the trophoblasts and delivered to the embryo fluid (EF) to modify the embryonic transcriptome.

MicroRNA-30d acts as a crucial coordinator for embryo-maternal cross-talks and regulates plenty of gene expressions involved in human embryonic implantation. Analyzing miRNA expression patterns showed significant upregulation of miR-30d in the epithelial layer of receptive human endometrium in the early mid-secretory phase versus pre-receptive endometrium (Altmäe et al., 2013; Moreno-Moya et al., 2014). Human endometrium secretes free form or exosome-coated miR-30d in the embryo fluid (EF), where the pre-implantation embryo can take it up *via* the trophectoderm layer (Figure 1). Also, treating mice embryos with miR-30d resulted in increased embryo adhesion rate, whereas using a specific miR-30d inhibitor led to a reduced adhesion rate. Additionally, the adhesion molecules such as integrin beta-3, integrin alpha-7, and cadherin-5 were upregulated following mir-30d treatment (Vilella et al., 2015). It was also shown that miR-30d in mice and humans had the same targets to extend the results to humans (Vilella et al., 2015).

Balaguer et al. showed the correlation between maternal miR-30d deficiency and diminished endometrial receptivity markers, which led to decreased implantation rates and impaired fetal development in mice (Balaguer et al., 2019). Furthermore, miR-30d transfection in the epithelial cell line of human endometrium induced the expression of abundant mRNAs and proteins associated with embryo adhesion, implantation, and development (Moreno-Moya et al., 2014).

In another study in 2021, it was demonstrated that miR-30d-5p expression levels were considerably decreased in women with RIF compared to average fertile women (Zhao et al., 2021). miR-30d-5p is also possibly responsible for SOCS1 downregulation, which ultimately inhibits the activation of the LIF-induced STAT3 pathway. In addition to embryo adhesion, miR-30d enhances endometrial angiogenesis by inhibiting MYPT1 and consequent VEGF activation (Lin et al., 2017; Zhao et al., 2021). Therefore, the impact of miR-30d and associated pathways on implantation can improve the outcome.

### 2.4 Adhesion, invasion, and angiogenesis associated signaling pathways

Upregulation of specific molecular pathways involved in human embryo implantation has been detected in WOI. Adhesion molecules such as integrin beta-3, cadherin-5, L-selectin, and mucin 1 (MUC-1) play critical roles in apposition and adhesion. Growth factors such as insulin-like growth factor 1 (IGF), heparin-binding epidermal growth factor (HB-EGF), and vascular endothelial growth factor (VEGF) are essentially required to develop a normal vascular network. Additionally, inflammatory responses enhance endometrial receptivity during WOI through inflammatory cytokines, including IL-1, IL-6, and leukemia inhibiting factor (LIF), which regulate fetal-maternal interactions during pregnancy and are essential for embryo implantation, trophoblast growth, and differentiation (Trolice and Amyradakis, 2012). LIF/gp130/STAT pathway is also crucial for embryo implantation as it is pivotal in angiogenesis. LIF acts through activation of gp130/STAT3 signals and subsequent VEGF expression. LIF glycoprotein expression level increases during the WOI in the secretory phase of normal fertile women. On the contrary, in RIF patients, the LIF level in the secretory phase was decreased (Hambartsoumian, 1998). Furthermore, endometrial and blood levels of LIF were concordantly decreased in RIF patients, followed by pregnancy failure (Comba et al., 2015).

There was also a significant decrease in both protein and mRNA levels of LIF and gp130 in most women with unexplained infertility and RIF at the proliferative and the secretory phases (Hambartsoumian, 1998; Tawfeek et al., 2012; Zhao et al., 2021). Heterozygous mutation of the LIF gene results in reduced LIF activity and may be the infertility cause in some women (Giess et al., 1999). Moreover, LIF/JAK/STAT3 pathway is involved in angiogenesis in the endometrium and placenta *via* inducing VEGF expression. LIF also positively impacts integrin expression, and VEGF *per se* induces integrin expression, so they are crucial in inducing the adhesion (Alfer et al., 2017) (Figure 1).

As shown in Figure 1, activation of LIF/JAK/STAT3 is also involved in promoting invasion *via* increasing the expression of MMPs (Suman et al., 2013). Upregulation of the JAK/STAT3 pathway negatively impacts SOCS1 expression as well. SOCS1 expression is increased in the endometrium of the RIF patients. In a recent study in 2021, decreased LIF and p-STAT3 protein levels in the RIF patients were detected, which may represent the critical role of this pathway for successful embryo implantation. The negative correlation between SOCS1 and miR-30d-5p has been demonstrated in the mentioned study. Decreased levels of miR-30d in the RIF group reduce STAT3 and phosphorylation of JAK, while SOCS1 is significantly increased, representing that the SOCS1 gene is the target of miR-30d, which participates in embryo implantation (Zhao et al., 2021). The reduced angiogenesis by inhibition of the LIF/JAK/STAT3 pathway can be compensated by miR-30d upregulation. miR-30d is one of the angio-miRs and promotes angiogenesis *via* MYPT1/c-JUN/VEGFA pathway (Lin et al., 2017).

VEGF is an essential element in the embryo-endometrium reciprocal interactions. VEGF promotes vascularization and improves blastocyst adhesion, implantation, and growth capability. Furthermore, endometrial angiogenesis is stimulated by the embryo *via* the production of active VEGF-A, which can induce vessel formation and consequent placental development. It has been shown that VEGF-A gene mutation leads to embryo loss and impaired placental development, and other developmental anomalies (Carmeliet et al., 1996; Ferrara et al., 1996; Kapiteijn et al., 2006; Guo et al., 2021). VEGF gene polymorphisms correlate with an increased risk of RIF (Jung et al., 2016; Shim et al., 2018). It has also been reported that VEGF expression was significantly reduced in women with RIF (Hannan et al., 2011; Lash et al., 2012). Therefore, impaired vascularity in the endometrium of the group with unexplained subfertility during the mid-late follicular phase has been demonstrated (Raine-Fenning et al., 2004). This diminished blood perfusion may lead to pathological hypoxia, impairing endometrial receptivity in infertile patients. However, 2%–5% oxygen concentration at the initial steps of embryo development is known as physiological hypoxia, and it is not life threatening.

“Hypoxia-inducible factor 1 (HIF-1)” is overexpressed in tissues with low oxygen concentrations and acts as a hypoxia-sensitive transcription factor. HIF-1 modifies cell adaptation to hypoxic conditions as it is upregulated even in basic levels of physiological hypoxia (Moeinabadi-Bidgoli et al., 2021). Additionally to normal embryo development, which occurs in a physiologic hypoxic environment, HIF-1 also improves embryo’s survival in pathological hypoxic environments (Dunwoodie, 2009). Moreover, endometrial HIF-1α expression is upregulated in RIF women, owing to the possible hypoxic microenvironment in these patients’ endometrium (Xu et al., 2011; Chen et al., 2016). This overexpression possibly promotes local angiogenesis to overcome hypoxia-induced consequences. Studies have shown that vascularization at implantation sites is promoted by HIF-1α expression *via* inducing VEGF gene expression (Guo et al., 2021). Eventually, error-free function of the implantation-related signaling pathways seems indispensable for a successful pregnancy.

### 2.5 Treatment of embryo implantation failure using miR-30d

In 2019, (Balaguer et al., 2019) demonstrated that the miR-30d deficiency negatively impacts endometrial receptivity and fetal growth. There was a significant difference in mRNA levels of receptivity markers such as COX2, LIF, MSX1, MSX2, estrogen, and progesterone just in the early stages of implantation in miR-30d deficient murine compared to the wild type (WT). miR-30d deficiency during the WOI lowers the expression of adhesion molecules in the blastocysts such as integrin beta-3, integrin alpha-7, and cadherin-5 and subsequently impairs embryo implantation. Additionally, impediment of maternal miR-30d transfer to the embryo led to a decreased implantation rate. The absence of miR-30d also led to fetal and placental development impairment. They showed that the pretreatment of miR-30d knocked out (KO) embryos with miR-30d analogs recovered impaired implantation in both WT and KO groups (Balaguer et al., 2019). Also, transiently miR-30d transfection into *in vitro* cultured human endometrial epithelial cells (hEECs) positively activated genes associated with the reproductive system’s function (Moreno-Moya et al., 2014). So, using miR-30d seems to help improve the implantation outcome. miR-30d can be transferred *via* exosomes to reach the implantation microenvironment (Liang et al., 2017).

### 2.6 Exosomes as a transfer vehicle

As mentioned earlier, extracellular vesicles (EVs) are involved in the reproductive process, including implantation and embryo development. Exosomes are small EVs found in both uterine fluid and embryo culture mediums (Andronico et al., 2019). Indeed, there is a bidirectional embryo-maternal communication through these secreted exosomes, whereby endometrial cells and the embryo take up one another’s exosomes (Figure 1). Meanwhile, endometrial exosomes are reported to be intensely engaged in facilitating the optimized condition for embryo implantation. (Blázquez et al., 2018) demonstrated that EVs derived from endometrial mesenchymal cells (EV-endMSCs) positively impacted embryo total cell number, development, and hatching (Blazquez et al., 2018). As mentioned before, EV miRNAs are essential regulators of the implantation process. In fact, exosomes are delivery vehicles for transferring miRNAs (Andronico et al., 2019). Due to exosomes’ limited size capacity, hybrid exosomes were used to load larger plasmids in a recent study (Lin et al., 2018). Afterward, these hybrid exosomes are capable of carrying CRISPR/dCas9 as a potential option to regulate miR-30d expression (Lin et al., 2018).

### 2.7 Targeted gene overexpression *via* CRISPR/dCas9; using exosome–liposome hybrid nanoparticles

Activation of the miR-30d gene *via* CRISPR/dCas9 can be considered a novel treatment for implantation impairment. Nuclease-deactivated Cas9 (dCas9) can be used for cellular re-programming, such as activating silent endogenous genes or over-expression and down-expression of specific genes. Since dCas9 endonuclease activity is suppressed, it is an effective gene-editing tool for activating or repressing gene expression without breaking the DNA. CRISPR/dCas9 can be a more powerful activator *via* fusing dCas9 proteins to a tetramer of VP16 to make a hybrid protein called dCas9‐VP64 (Chavez et al., 2015). This hybrid combination requires a suitable transfer system as well. There are many targeted gene delivery systems, including viruses, liposomes, and membrane-derived vesicles such as exosomes. In order to avoid triggering immunogenic responses, exosomes will be administered. Delivery of the CRISPR/dCas9 with exosome–liposome hybrid nanoparticles is an effective way to transfer large-sized plasmid DNA and overcome the small size of exosomes (Lin et al., 2018; Nazerian et al., 2021).

## 3 Strategy

**FIGURE 2:**
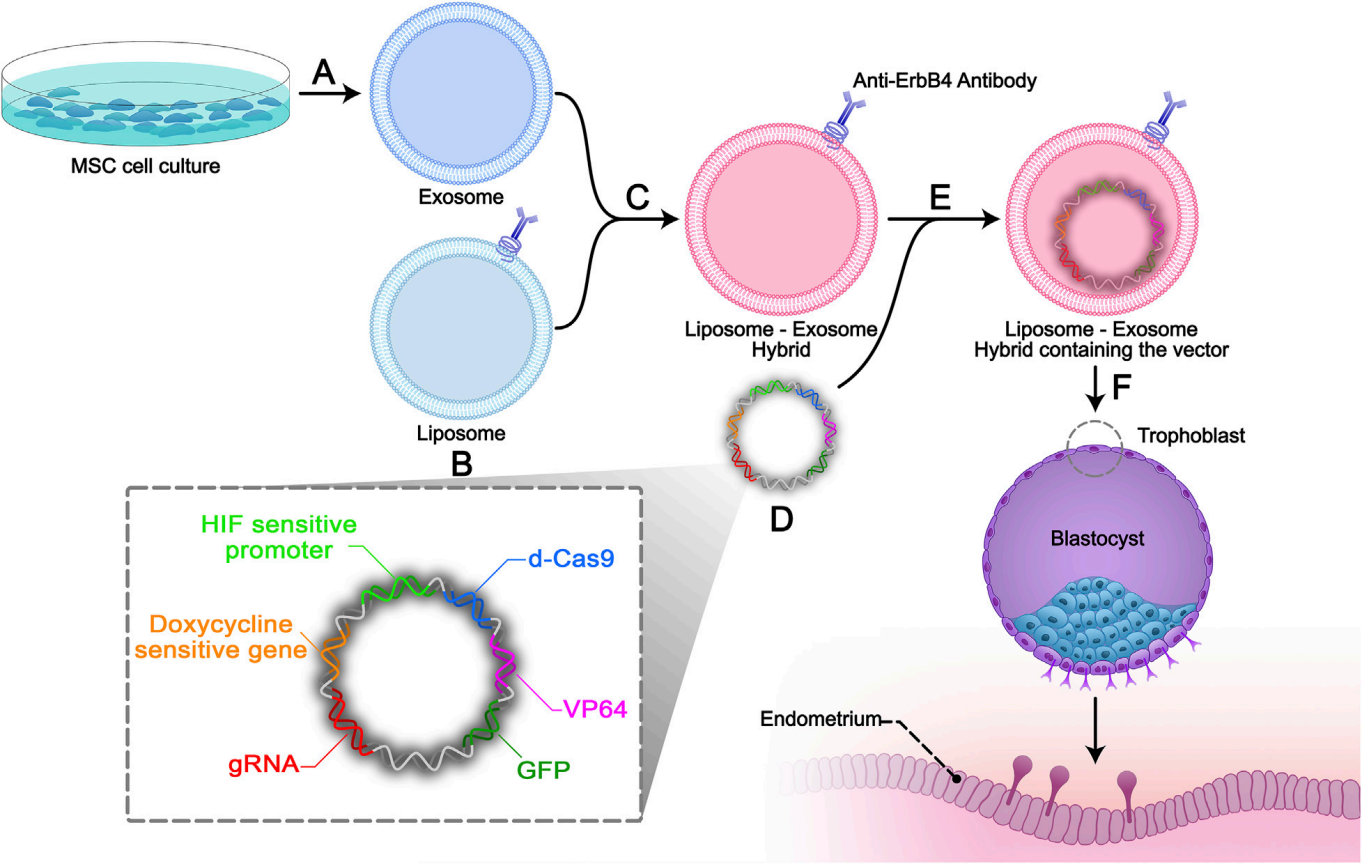
Study design. **(A)** Isolation and characterization of Mesenchymal stem cell-derived exosomes (MSC exosomes) by the ultra-centrifuge method. **(B)** Antibodies against ErbB4 conjugate on the surface of constructive liposomes. **(C)** Exosomes derived from end-MSCs will be incubated with the targeted liposomes to make exosome-liposome hybrids. **(D)** Non-viral S/MAR episomal vector will be constructed containing: sgRNA, HIF-1 sensitive promotor (evidence), dCas 9, VP64, GFP gene sequences, doxycycline sensitive gene, and ribosomal skipping 2A sequences between the genes. **(E)** Electroporation will be used to encapsulate the designed episomal S/MAR vector into the hybrid. **(F)** The liposome-exosome hybrids will be added to the trophoblast cell media and the episomal vector will specifically enter the trophoblast.

We suggest that the hypoxia-sensitive regulation of miR-30d expression *via* trophoblast-specific dCas-9 delivery by engineered exosomes could enhance adhesion, invasion, and angiogenesis, leading to improved implantation and bypass placental insufficiencies during pregnancy. This strategy consists of three components:

(1) It is possible to utilize miR-30d as an upstream transcriptomic regulator of the pre-implantation embryo, leading to overexpression of specific encoding genes involved in embryo adhesion, invasion, and angiogenesis. Upregulation of certain implantation involved molecules, including VEGF, endometrial estrogen/progesterone receptors, LIF, adhesive proteins such as ITGA7, ITGB3, and CDH5 alongside COX-2 enzyme production are caused by miR-30d (Vilella et al., 2015; Balaguer et al., 2019; Kolanska et al., 2021). Moreover, studies have shown that miR-30d deficiency during preconception leads to impaired endometrial receptivity and fetus development (Balaguer et al., 2019).
(2) It is possible to enter the deactivated CRISPR/dCas9 as a miR-30d gene activator into trophoblasts. In addition to the lower cost, the advantage of dCas9 over gene entry into the trophoblasts is the presence of single guide RNA (sgRNA), which makes miR-30d gene activation explicitly targeted. Moreover, dCas9 is preferable to other gene activation methods, including drugs, due to its stability in trophoblasts during pregnancy and the possibility of gene expression regulation by regulatory promoters (Brezgin et al., 2019). To prevent dCas9 harmful overactivity, we can utilize regulatory promoters containing hypoxia-response elements (HREs) as a binding site for hypoxia-inducible factor 1 (HIF-1), located upstream of dCas9 (Javan and Shahbazi, 2017; Shakirova et al., 2020) (Figure 2). Thus, before and during implantation and in conditions of placental insufficiency, the miR-30d expression increases and will be extinguished after hypoxia elimination.
(3) As an ideal gene therapy vector, a plasmid vector containing a “scaffold/matrix attachment region (S/MAR)” domain is capable of persistent expression activity with much fewer safety concerns in comparison with other common vectors such as lentiviral vectors. The non-viral S/MAR vectors can replicate episomally as extra-chromosomal entities in targeted trophoblasts (Wong et al., 2011; Bozza et al., 2020). Among various dCas9 delivery carriers, the advantage of exosomes is their low immunogenicity (Samanta et al., 2018). However, due to the limitation of exosome size, it is suggested to utilize exosome-liposome hybrids as carrier (Biagioni et al., 2018). As shown in Figure 2, these hybrid exosomes are equipped with ErbB-4 ligand on their surface in order to exclusively enter the trophoblast layer without infecting the inner cell mass.

## 4 Evaluation of strategy

In order to evaluate the proposed strategy of regulating miR30-d expression *via* trophoblast-specific dCas9 delivery, *in vitro* and *in vivo* experimental studies would be conducted. First, in the *in vitro* section, an episomal vector containing dCas9 and complementary sgRNA of the miR-30d gene is designed and encompassed within a specific exosome-liposome hybrid. This hybrid carrier should be dressed with a trophoblast-specific antibody on its surface. Second, this hybrid carrier will specifically transfect the mouse blastocysts’ trophoblast cells, which were previously achieved from IVF. Finally, the implantation rate of this blastocyst will be evaluated *in vitro*. In the *in vivo* section, these transfected blastocysts will be transferred to the female mice uterus, and the implantation rate will be evaluated *in vivo* (Figure 2).

### 4.1 *In vitro* studies

#### 4.1.1 Exosome extraction of endometrial mesenchymal stem cells

Endometrial mesenchymal stem cells (endMSCs) can be isolated from the menstrual blood of healthy women (Moreno et al., 2017). Exosomes from endMSCs is extracted by the exosome isolation medium and further centrifugations (Blazquez et al., 2018).

#### 4.1.2 Trophoblast-targeted liposomes designing

Liposomes are widely used for gene and drug delivery purposes. Lipoplexes stemming from cationic liposomes generally possess high cargo delivery efficacy *in vitro* and form multilamellar structures with multiple bilayers, and CRISPR/dCas9 are contained within lipid bilayers (Zhen and Li, 2020). It has been demonstrated that non-specific ionic interaction between the liposome/lipoplexes cationic surfaces with the anionic lipid surface of the cell membrane and proteoglycans contributes to the cellular uptake process (Zhang et al., 2004; Zhen and Li, 2020). Moreover, the ErbB4 targeting ligand is attached to the liposome surface to improve further cellular absorption as well as site-specific gene delivery (Paul et al., 2017). Surface functionalization of liposomes by attaching targeting ligands on the outer lipid layer can make an active targeting liposome (Riaz et al., 2018). Therefore, after constructing liposomes, antibodies against ErbB4, a specific surface protein of the trophoblast, will be conjugated on the surface of these liposomes (Chobotova et al., 2002). The remedy to produce cationic lipoplexes is described in detail in previous studies such as by Junquera and Aicart (2016).

#### 4.1.3 Liposome-exosome hybrid production

Exosomes are small-sized membrane vesicles that can carry different kinds of molecules. Because of their small size, hybrid exosomes are promising carriers for the targeted and efficient delivery of desired molecules (Lin et al., 2018). In order to make hybrid exosomes, the derived exosomes from endMSCs will be incubated with the surface-targeted liposomes at 37 C for 12 h, as shown by Lin et al. (2018). In this study, we suggested the incubation route due to its simplicity and efficacy in preserving the exosome and liposome membranes. After that, our plasmid vector can be encapsulated into this hybrid. The fusion of liposomes and exosomes will be confirmed by dynamic light scattering (DLS) for size distribution plus zeta potential (ZP) analysis. DLS measures the hydrodynamic diameter of hybrid exosomes by the Stokes-Einstein equation. Besides larger size, hybrid exosomes often possess slightly reduced ZP compared to liposomes due to the initial negative charges of the exosomes (Bhattacharjee, 2016). The following exosomal imaging will reveal confirmation of the fusion (Bhattacharjee, 2016; Liu et al., 2022). Flow cytometry and western blot analysis will further confirm the anti-ErbB4 ligand’s existence on the surface of liposome-exosome hybrid extracts (Lin et al., 2018; Alghuthaymi et al., 2021). Also, the surface topology of the ErbB4 antibody on liposomes will be determined by calculating the incorporation ratio as described by for post-inserted liposomes Lee et al. (2016).

Since our hybrid exosomes are transported to the trophoblast membrane *in vitro*, there is no need for surface PEGylation regarding increasing the circulation stability; especially due to the decreased cellular uptake and endosomal escape ratio following PEGylation.

#### 4.1.4 Episomal vector design and delivery into the liposome-exosome hybrid

S/MARs are AT-rich DNA sequences that bind chromatin to the nuclear matrix (Stavrou et al., 2019). S/MAR vectors are non-integrating non-viral episomal vectors with low immunogenicity (Wong et al., 2011). As shown in Figure 2, our non-viral S/MAR episomal vector contains sgRNA, HIF-1 sensitive promoter, dCas 9, VP64, GFP gene sequences, doxycycline sensitive gene, and ribosomal skipping 2A sequences between the genes (Xie et al., 2017). In order to encapsulate the designed episomal S/MAR vector into the hybrid carrier, electroporation will be conducted (Bunggulawa et al., 2018).

#### 4.1.5 IVF procedure

Oocytes from the female BDF mouse model will be obtained after ovulation stimulation by administering the pregnant mare serum gonadotropin. 48 h later, human chorionic gonadotropin (hCG) will be administered and stored in the appropriate media. Sperms of the male rats will be collected from the end of their epididymis and, after capacitation and maturation, will be added to the oocytes to fertilize them. During its development, the zygote will further form the trophoblast and the inner cell mass layer (Balaguer et al., 2019).

#### 4.1.6 Episomal vector transduction into the trophoblast cells

In order to deliver the genes specifically into the trophoblast cells, the liposome-exosome hybrids with the trophoblast-specific antibodies on their surface will be added to the trophoblast cells media. By attaching the anti-ErbB4 antibodies on the surface of the hybrid carrier to the ErbB4 protein on the trophoblast surface, the episomal vector will enter the trophoblast exclusively. In addition, the trophoblast layer covers the inner cell mass and have a protective role against foreign molecular or cellular components, including exosomes (Georgiades et al., 2007). Besides, western blot analysis and fluorescent imaging will be used for tracking the GFP protein expression to confirm the specific transduction of the desired vector to the trophoblast cells (Okada et al., 2007).

#### 4.1.7 miR-30d expression measurement in the transfected blastocysts

After transfection of the blastocysts, the efficiency of this delivery system and miR-30d expression level will be assessed by the RT-PCR method.

#### 4.1.8 Evaluation of the effect of miR-30d overexpression on the implantation rate

Two groups will be needed to evaluate the effects of mir-30d overexpression on the implantation. Normal blastocysts obtained from the IVF procedure without any gene manipulation will be co-cultured with endMSC-derived exosomes in the first group. The second group will consist of the blastocysts transfected with the episomal vector in the zygote stage and cultured in the same conditions as the first group. The number of attached embryos in both groups will be compared before and after shaking on a rotation shaker to assess the implantation rate.

In order to determine the changes in the molecular level of the implantation, attached embryos will be separated from the endometrial cells, and the level of CDH5, ITGB3, and ITGA7 proteins and gene expression in the embryos will be assessed by Western blot analysis and RT-qPCR, respectively. Additionally, Western blot analysis and RT-qPCR will assess the level of LIF, VEGF, and COX2 proteins and gene expression in the endometrial cells, respectively (Vilella et al., 2015).

### 4.2 *In vivo* studies

Five groups will be considered to evaluate the efficacy of enhancement in the miR-30d expression on the implantation: 1- The first group consists of the female mice that mate with the male mouse and will be pregnant. 2- The second group consists of the female mice housed with vasectomized males. 3- The third group consists of female mice that receive blastocyst of IVF procedure without any gene manipulation. 4- The fourth group consists of female mice that received blastocyst of IVF procedure and were incubated with the liposome-exosome hybrids lacking episomal vectors. 5- The last group consists of female mice that receive blastocyst of IVF procedure that were genetically manipulated by trophoblast-specific gene delivery as discussed previously. 4 days before embryo transfer (ET), in the third, fourth, and the fifth groups, female mice will be housed with vasectomized males to become pseudopregnant, which is necessary for pregnancy. Then ET will be performed by the non-surgical embryo transfer method.

#### 4.2.1 Evaluation of the effects of dCas9 gene delivery on the *in vivo* expression of miR-30d

Oviduct of the mice in each group will be dissected on days 6, 12, and 16 of their pregnancy. Then, the RT-qPCR method will assess the miR30d expression level in the trophoblast cells. Besides, confirmation of specific gene delivery into the trophoblast layer and not in the inner cell mass layer will be revealed by fluorescence microscopic imaging by tracking the GFP.

#### 4.2.2 *In vivo* evaluation of the increase in miR-30d expression on the implantation

On days 6, 12, and 16 of the pregnancy, implantation sites are counted after intravenous injection of the Chicago Sky blue solution. In addition, on days 6, 12, and 16 of pregnancy, mice will be sacrificed, the uterus and placenta will be isolated, and the uterine and placental levels of LIF, VEGF, integrin alpha-7, integrin beta-3, and COX2 will be assessed at the protein and gene level by Western blot and RT-qPCR techniques, respectively. Fetal growth restriction and placental insufficiency will be evaluated by measuring crown-rump length (CRL), the embryos' weight, and the placenta’s weight on days 12 and 16 of pregnancy.

## 5 Discussion and future direction

Implantation failure is the major limitation in ART procedures and mostly stems from impaired endometrial receptivity (Franasiak et al., 2014; Craciunas et al., 2019). Improving endometrial receptivity leads to enhanced embryo adhesion, invasion, and angiogenesis as the crucial steps of embryo implantation. Considering the critical role of miR-30d in regulating endometrial receptivity by activating related signalling pathways such as LIF/gp130/STAT and upregulation of VEGF, we proposed that the miR-30d upregulation will assist the blastocyst with the implantation. Besides, miR-30d will improve the fetus’s survival in response to low oxygen concentrations during pregnancy. In low oxygen conditions, the HIF transcription system is activated and helps the embryo survive the harsh hypoxic environment. HIF-1α is mainly associated with hypoxic conditions (Dunwoodie, 2009). Therefore, a controlled increase of miR-30d in a hypoxia-sensitive manner in response to HIF-1α upregulation is suggested in this study.

As previous studies demonstrated, miRNA expression patterns alter at each stage of the female menstrual cycle and are also differentially expressed in fertile women compared with RIF patients (Rekker et al., 2018; von Grothusen et al., 2022). Meanwhile, the miR-30d expression level undergoes the most significant changes compared to other microRNAs during the time of WOI, indicating the potentially pivotal role of miR-30d on embryo implantation (Kuokkanen et al., 2010; Vilella et al., 2015). Endometrial secreted miR-30d is absorbed by the trophectoderm cells to modify the gene expression of the pre-implantation embryo (Altmäe et al., 2013; Moreno-Moya et al., 2014; Vilella et al., 2015). One of these gene modifications is inducing VEGF expression by inhibiting MYPT1 and activating the LIF-induced STAT3 pathway to promote endometrial angiogenesis. MiR-30d promotes the activation of the LIF-induced STAT3 pathway, possibly *via* downregulating SOCS1 in the embryo and endometrium (Lin et al., 2017; Zhao et al., 2021). Impaired angiogenesis due to decreased VEGF levels is extensively detected in RIF patients (Hannan et al., 2011; Lash et al., 2012). Endometrium-derived and embryo-derived VEGF are involved in endometrial angiogenesis, increased implantation success, and placental development. Besides angiogenesis, LIF-induced pathways promote adhesion and invasion by inducing integrins’ expression and MMPs, respectively (Alfer et al., 2017; Guo et al., 2021; Zhao et al., 2021). So, the activation of the LIF/JAK/STAT3 pathway promotes angiogenesis by inducing VEGF expression, the invasiveness of trophoblastic cells by activating the transcription of MMPs, and adhesion by increasing the expression of integrins. In conclusion, regulating miR-30d expression is presumed to facilitate the implantation process in RIF patients by enhancing adhesion, invasion, and angiogenesis.

There have also been reports of the miR-30d upregulation effects on the human endometrial endothelial cells (hEECs) and mice models, resulting in the activation of reproduction-related genes and improved implantation outcomes (Moreno-Moya et al., 2014; Vilella et al., 2015). Also, (Balaguer et al., 2019) assessed the impact of miR-30d deficiency on endometrial receptivity and reported a significantly lower implantation success rate in mir-30d knocked-out mice models compared to wild-type and miR-30d-treated ones (Balaguer et al., 2019). Therefore, miR-30d upregulation seems imperative for a successful pregnancy, although the results of the previous studies were limited to the implantation phase and were not in a controlled manner. In our proposed strategy, miR-30d expression is consistently regulated through a feedback mechanism following hypoxic stress and comes up with a more controlled outcome. Besides improving implantation, the hypothesized strategy aids in placental insufficiency during the later stages of pregnancy.

In order to control miR-30d expression, an episomal vector containing mainly CRISPR/dCas9 and a miR-30d promoter sgRNA is suggested in this study. Utilizing CRISPR/dCas9 instead of CRISPR/Cas9 for gene delivery due to dCas9 incapability of breaking the host DNA can considerably reduce the risk of damaging the embryo DNA. Genetic manipulation of embryos has been performed in several previous studies have used direct co-injection of CRISPR/Cas and sgRNA to generate mutations in rats (Balaguer et al., 2019). However, their study’s CRISPR/dcas9 delivery method is different from ours, but they demonstrated efficient gene editing in embryos followed by a successful pregnancy. Another study by (Tröder et al., 2018) reported successful genome editing by entering CRISPR/Cas into mouse zygotes *via* electroporation (Tröder et al., 2018). Regardless of CRISPR/Cas delivery method variations in previous studies, the use of CRISPR/Cas in gene editing led to desired genetically engineered births. Nevertheless, the uncontrolled expression of miR-30d could be alarming for the mother and the fetus’s health. Hence, a regulatory promoter containing hypoxia-response elements (HREs) is used as a binding site for hypoxia-inducible factor 1 (HIF-1) to control miR-30d gene expression in the absence of hypoxic stress. Besides, the doxycycline susceptibility gene will be inserted into the designed vector to function as an off switch if required. This strategy minimizes the risk of inadvertent miR-30d overexpression.

As for vector delivery, a liposome-exosome hybrid can transfer larger nucleic acid fragments compared to common exosomes (Lin et al., 2018; Duan et al., 2021). For the liposomal component, cationic lipoplexes are sufficiently qualified for CRISPR delivery due to the enhanced encapsulation of negatively charged nucleic acid by electrostatic attraction. Additionally, cationic liposomes have a better chance of entering the trophoblast membrane through interacting with anionic surface lipids and proteoglycans (Zhen and Li, 2020; Nsairat et al., 2022). The liposome lipoplexes’ cationic lipids form ionic bonds with the endosomal anionic membrane, which eventually results in lipoplex disassembly and the release of CRISPR content of the liposomes into the cytoplasm (Kazemi Oskuee et al., 2016). For safe gene delivery, we will equip the liposomal surfaces with Erb-B4 targeting ligands to precisely aim for the trophoblast cells’ surface to surmount the concern of infecting the inner cell mass (Takeuchi et al., 2017; Wood et al., 2021). On the plus side, the surrounding trophectoderm layer prevents the passage of large molecules and cell particles, including exosomes, into the inner cell mass (Hemberger et al., 2020). Targeted therapy of miR-30d overexpression provides considerable advantages, including exclusive gene delivery to trophoblast cells, restrained undesired effects of blastocyst genetic manipulation, and fewer administered hybrid exosomes needed.

Finally, miR-30d can be used as a therapeutic option for increasing embryo implantation success. This study offers a therapeutic strategy based on transferring hypoxia-sensitive episomal vectors *via* trophoblast-specific dCas9 delivery using engineered hybrid exosomes. In the pathologic hypoxic conditions, the miR-30d expression would increase due to the binding of HIF-1α to the HREs promoter. The elevated expression of miR-30d leads to increased endometrial receptivity resulting in enhanced embryo adhesion, invasion, and angiogenesis. Besides improving implantation, this strategy would hypothetically make the fetus capable of surviving the harsh environment caused by pathologic hypoxia for the rest of the pregnancy.

As far as we know, no previous studies have administered hypoxia-sensitive miR-30d regulation through HIF-1α expression to improve the implantation success rate. Therefore, it is crucial to investigate this therapeutic strategy in upcoming ART studies.

## Data Availability

The original contributions presented in the study are included in the article/supplementary material, further inquiries can be directed to the corresponding author.
